# Pancreatic shear wave elastography in children with type 1 diabetes: relation to diabetes duration, glycemic indices, fasting C-peptide and diabetic complications

**DOI:** 10.1007/s00247-022-05363-1

**Published:** 2022-04-23

**Authors:** Nouran Yousef Salah, Sherihane Said Madkour, Khaled Sayed Soliman

**Affiliations:** 1 Pediatrics Department, Faculty of Medicine, Ain Shams University, Qorash Street, Nasr City, Cairo, 11765 Egypt; 2 Radiology Department, Faculty of Medicine, Ain Shams University, Cairo, Egypt

**Keywords:** C-peptide, Children, Diabetes Complications, Elastography, Pancreas, Type 1 diabetes, Ultrasound

## Abstract

**Background:**

Little is known about changes in the pancreas as the course of type 1 diabetes progresses. Recently, shear wave elastography (SWE) emerged as a tool for assessing pancreatic stiffness in chronic pancreatitis and pancreatic cancer with a few studies assessing it in diabetes.

**Objective:**

To compare pancreatic SWE in children with recent-onset and long-standing type 1 diabetes to healthy controls and to correlate it with diabetes duration, glycated hemoglobin (HbA1C), functional B cell reserve (fasting C-peptide) and diabetic complications.

**Materials and methods:**

Fifty children with type 1 diabetes (25 with recent-onset and 25 with long-standing type 1 diabetes) and 50 controls were enrolled. Diabetes duration, insulin therapy, fundoscopic examination of the eyes and the neuropathy disability score were assessed. Fasting C-peptide, lipids, HbA1C and urinary albumin-creatinine ratio were measured. Pancreatic SWE was measured using the General Electric Logiq P9 ultrasound system.

**Results:**

The mean SWE of the studied children with recent-onset type 1 diabetes was 4.81±0.62 kilopascals (Kpa), those with long-standing type 1 diabetes was 7.10±1.56Kpa and for controls was 5.57±0.27 Kpa (*P*<0.001). SWE was positively correlated to diabetes duration (*P*<0.001) and negatively correlated to fasting C-peptide (*P*<0.001). Regarding diabetes complications, SWE was positively correlated to frequency of severe hypoglycemia (*P*=0.005), HbA1C (*P*=0.03), low-density lipoproteins (*P*<0.001) and cholesterol (*P*<0.001) and significantly related to diabetic neuropathy (*P*=0.04) and nephropathy (*P*=0.05). Diabetes duration, fasting C-peptide, HbA1C and frequency of severe hypoglycemia were the significant independent variables related to SWE increase by multivariable regression analysis.

**Conclusion:**

Pancreatic SWE changes significantly with duration of type 1 diabetes, being lowest in those with recent-onset type 1 diabetes and highest in those with long-standing type 1 diabetes, particularly those with diabetic nephropathy and neuropathy.

## Introduction

Type 1 diabetes is a chronic disease characterized by autoimmune destruction of the insulin-producing beta cells of the pancreas resulting in absolute insulin deficiency [1]. Different triggers of β-cell autoimmunity cause local pancreatic-islet inflammation (insulitis), which eventually leads to a decrease in the size of the pancreas and functional β-cell mass [2, 3]. People with type 1 diabetes genetic risk alleles have a hyper-inflammatory immune system and smaller pancreatic size than unrelated controls [4]. Yet the relationship between pancreatic pathology and disease progression is still obscure.

Islets of patients with recent type 1 diabetes diagnosis show insulitic lesions defined as six or more CD3+ (cluster of differentiation 3 positive) cells within the pancreatic islet with three or more islets affected per pancreas section, while islets of patients with long-standing diabetes no longer show insulitis [5]. Insulitis in people with type 1 diabetes genetic risk alleles and those with recent-onset type 1 diabetes and its absence in long-standing type 1 diabetes might reflect prodromal islet distress and prediabetic lesions. However, little is known about the corresponding pancreatic changes during the progression to type 1 diabetes since pancreatic islets imaging is not feasible in vivo and pancreatic biopsies, which would enable longitudinal studies of the changes of islet cell composition and cell-cell interactions, are not undertaken due to their invasiveness [6].

C-peptide is the part of proinsulin cleaved before co-secretion with insulin from pancreatic beta cells. It is produced in equimolar amounts to endogenous insulin but is not a product of therapeutically administered exogenous insulin. It is widely used as a measure of insulin secretion and pancreatic beta cell function [7].

Elastography is a noninvasive imaging modality that assesses tissue elasticity and stiffness [8]. Two types of elastography exist: strain elastography and shear wave elastography (SWE). SWE induces mechanical waves, including shear waves, which propagate transversely in the tissue. It assesses the propagation speed of these shear waves within a given region of interest (ROI) quantifying stiffness in kilopascals (Kpa) or meters per second (m/s). The faster the shear wave propagates, the stiffer the tissue. SWE is more reproducible and less operator dependent than strain elastography [9]. The procedure was initially introduced for the assessment of liver fibrosis [10]. Later, it was used to assess other tissues including the breast and thyroid gland [11, 12]. SWE is useful in differentiating tissue inflammation from fibrosis, owing to the difference in tissue stiffness, being low in inflammation and high in fibrosis [13–15].

Recently, a few studies have used this technique to assess pancreatic tissue [16, 17]. Shear wave velocity (SWV) was found to be positively correlated with the histological grade of pancreatic fibrosis and stiffness in adults with chronic pancreatitis and pancreatic cancer [– 18, 20]. In addition, a few studies have assessed pancreatic shear wave elastography in adults with type 1 diabetes and Type 2 diabetes [1, 21].

Hence, this study aims to assess pancreatic shear wave elasticity in children with recent-onset and long-standing type 1 diabetes in comparison to healthy controls and to correlate it with diabetes duration, functional B cell reserve and microvascular complications.

## Materials and methods

### Study design

This cross-sectional study included 50 children with type 1 diabetes and 50 age- and gender-matched healthy controls. Type 1 diabetes was defined according to the criteria of the International Society of Pediatric and Adolescent Diabetes [22]. Exclusion criteria included patients with other types of diabetes, e.g., type 2 diabetes and maturity-onset diabetes of the young, patients with obesity, and patients with comorbid autoimmune disorders that might cause chronic pancreatitis (systemic lupus, autoimmune pancreatitis). Children with type 1 diabetes were recruited consecutively from the Pediatrics and Adolescents Diabetes Unit, Ain Shams University, while the controls were recruited from the outpatient clinic during the period March 2021 to September 2021. Children with type 1 diabetes were classified into two equal groups, those with recent-onset type 1 diabetes having diabetes for less than 6 months and those with long-standing diabetes having diabetes for more than 5 years. The study was approved by the research ethics committee of Ain Shams University**.** The study adhered to the tenets of the Declaration of Helsinki and all procedures were explained to all participants, with written informed consent obtained from them or their legal guardians.

### Clinical assessment

All included patients were subject to a full history including age, gender, family history of type 1 diabetes, diabetes duration, insulin therapy (basal bolus regimen or insulin pump and total daily dose) and history of chronic microvascular diabetic complications (retinopathy, neuropathy and nephropathy).

Clinical examination included anthropometric measures (weight and height) with calculation of standard deviation score and body mass index (BMI) [23]. A specialized pediatric ophthalmologist “(not an author) with 12 years of experience performed fundal examination by direct ophthalmoscopy through dilated pupils to assess diabetic retinopathy [24].

The simple rapid bedside neuropathy disability score was adopted as a screening tool for diabetic peripheral neuropathy. The sensory modalities were scored; a score above two was defined as clinical diabetic peripheral neuropathy [25].

### Laboratory assessment

Fasting C-peptide, fasting lipids, HbA1C and urine albumin-to-creatinine ratio (UACR) were assessed in the studied children with type 1 diabetes. Fasting triglycerides and total cholesterol were performed by quantitative enzymatic colorimetric technique using a commercial kit purchased from Bio Merieux (Diagnostic Chemicals Ltd., Charlottetown, CA). High density lipoproteins (HDL) were measured by the phosphotungstate precipitation method using a Bio Merieux kit (Marcy-l’Etoile, Craponne, France). Results were clinically interpreted according to recommendations of the European Atherosclerosis Society [26].

Fasting C-peptide was assessed using Cobas e 411 (Roche Diagnostics, Mannheim, Germany) and HbA1C was assessed using D−10 (BioRad, Paris, France) [27]. UACR in an early morning fasting urine sample was measured by an immuno-turbidimetric method (Cobas Integra 800, Roche Diagnostics). Nephropathy was defined as the presence of micro- or macro-albuminuria [28].

### Radiologic assessment

Conventional B-mode and 2-D SWE examinations were performed using LOGIQ P9 GE ultrasound system (GE Healthcare, Milwaukee, WI) using a 3.5- to 5-Mhz convex ultrasound transducer for all patients and controls. Examinations were performed and interpreted by two experienced radiologists (S.S.M. with 11 years’ experience and K.S.S. with 10 years’ experience) blinded to each other and to patients’ clinical and laboratory findings. The radiologists assessed patients separately and the mean score of both radiologists’ readings was taken for each patient.

The ultrasound transducer was placed in the epigastric region to visualize the entire pancreas. The pancreas was demonstrated in B-mode first, then 2-D SWE was performed in elastography display mode allowing 2-D elastography to be obtained. The ROI was placed on the pancreatic body ensuring that no blood vessels, capsule or pancreatic duct was located within it. The body was distinguished from the head by the confluence of the splenic and superior mesenteric veins. The tail was defined as the structure opposite the medial margin of the left kidney extending to the hilum of the spleen (identified by the splenic artery and vein) [1, 29].

The SWE measurements were automatically reported by the ultrasound machine by measuring 12 readings of elasticity at the ROI and calculating their median. Unacceptable shear wave measurements based on location or artifact were repeated. Reliable shear wave measurements were those with interquartile range (IQR) less than 30%.

### Sample size

Sample size was calculated using the PASS11 program, setting the alpha error (α) at 5% using two-tail hypothesis testing. Reviewing results from a previous study by Öztürk and Yildirim [30] showing that elastography is a safe noninvasive and accurate method for early detection, long-term screening and follow-up of children with type 1 diabetes with area under the ROC curve (AUC) of 0.99, a sample size of 50 cases and 50 controls achieves 100% power to detect a difference in pancreatic elastography of 0.4900 between the AUC under the null hypothesis (0.5000) and an AUC under the alternative hypothesis (0.9900) using a two-sided z-test at a significance level of 0.05. The data are continuous responses. The AUC is computed between false-positive rates of 0.00 and 1.00. The ratio of the standard deviation of the responses in the patient group to the standard deviation of the responses in the control group is 1.00 [31, 32].

### Statistical analysis

Data were collected, revised, coded and entered into Statistical Package for Social Science (IBM SPSS) version 25. A Kolmogorov-Smirnov test was used to examine the normality of data. Quantitative data were presented as means, standard deviations and ranges when their distribution was parametric and median with IQR when their distribution was nonparametric. Qualitative data were presented as numbers and percentages. A one-way analysis of variance was used to compare parametric data sets. Nonparametric variables were compared using a Mann-Whitney test. A Tukey’s honestly significant difference post hoc test was performed if an overall significance was found. Qualitative variables were compared using a chi-square test. Spearman correlation coefficients were used to assess the correlation between two quantitative parameters in the same group. Before multiple linear regression analysis, several variables were log-transformed to obtain (approximate) normal distribution. Simple regression analysis was first performed to screen potential associations followed by a multivariate stepwise linear regression model to identify and determine significant associations for tissue stiffness and SWE. Using the approach of stepwise variable selection, stepping up, only variables with a significance level of 0.05 were included in the model. The confidence interval was set to 95% and the margin of error accepted was set to 5%. The *P*-value was considered significant at a level of <0.05.

## Results

Twenty-two males (44.0%) and 28 females (56.0%) with type 1 diabetes were studied. Their mean age was 13.04±3.39 years with a range of 2 to 18 years. Twenty-five subjects had recent-onset type 1 diabetes and 25 had long-standing type 1 diabetes. They were compared to 50 age- and gender-matched healthy controls (*P*=0.15 and *P*=0.32, respectively). All children with type 1 diabetes were on a basal bolus subcutaneous insulin regimen with a mean total daily dose of 1.18±0.30 U/kg/day. The clinico-laboratory data of the studied children with type 1 diabetes are listed in Table 1.

**Table 1:** Clinical, laboratory and radiologic data for children with type 1 diabetes

		Children with recent-onset type 1 diabetes *n*=25	Children with long-standing type 1 diabetes *n*=25	Test value	*P*-value^*d*^
Age (years)	Mean±SD	12.84±3.54	13.24±3.28	0.414^*a*^	0.68
	Range	2–14	10–18		
Disease duration (years)	Mean±SD	0.23±0.12	8.5±3.89	-6.207^*a*^	**<0.001**
	Range	0.1–0.3	5–16		
Gender	Females	11 (44.0%)	17 (68.0%)	2.922^*b*^	0.09
	Males	14 (56.0%)	8 (32.0%)		
Insulin dosage (U/kg/day)	Mean±SD	1.10±0.22	1.26±0.36	-1.923^*a*^	0.06
	Range	0.8–1.6	0.87–1.9		
HbA1C (%)	Mean±SD	9.75±1.16	9.91±2.57	−0.284^*a*^	0.78
	Range	7.5–11.2	7–13		
Fasting C-peptide (ng/mL)	Median (IQR)	0.2 (0.12–0.24)	0.05 (0.1–0.14)	4.046^*c*^	**<0.001**
	Range	0.03–0.9	0.01–0.2		
Frequency of severe hypoglycemia (per month)	Median (IQR)	1 (0–1)	1 (0–4)	2.389^*c*^	**0.02**
	Range	0–2	0–7		
LDL (mg/dl)	Mean±SD	85.44±14.15	114.32±25.81	−4.906^*a*^	**<0.001**
	Range	60–104	86–189		
HDL (mg/dl)	Mean±SD	52.72±14.98	50.83±10.59	0.515^*a*^	0.61
	Range	27–68	32–65.9		
Cholesterol (mg/dl)	Mean±SD	136.16±19.11	178.32±43.06	−4.475^*a*^	**<0.001**
	Range	120–181	135–285		
Triglycerides (mg/dl)	Mean±SD	112.92±52.15	111.15±54.42	0.117^*a*^	0.91
	Range	48–227	53–232		
UACR (mg/g creatinine)	Median (IQR)	12 (10–14)	31 (10–45)	2.145^*c*^	0.03
	Range	8–16	3.7–52		
Diabetic nephropathy	Negative	25 (100.0%)	12 (48.0%)	17.568 ^*b*^	**<0.001**
	Positive	0 (0.0%)	13 (52.0%)		
Diabetic neuropathy	Negative	25 (100.0%)	16 (64.0%)	10.976 ^*b*^	**0.001**
	Positive	0 (0.0%)	9 (36.0%)		
Diabetic retinopathy	Negative	25 (100.0%)	25 (100.0%)	NA	NA
	Positive	0 (0.0%)	0 (0.0%)		
Shear wave elastography (KPa)	Mean±SD	4.81±0.62	7.10±1.56	−4.287^*a*^	**<0.001**
	Range	3.87–5.9	4.62–9.5		

*HbA1C* glycated hemoglobin, *HDL* high-density lipoproteins, *LDL* low-density lipoproteins, *NA* not available, *SD* standard deviation, *UACR* urinary albumin creatinine ratio

*a*: Independent t-test; *b*: chi-square test; *c*: Mann-Whitney test; *d*: *P* value <0.05 is significant (bold)

### Pancreatic shear wave elastography in type 1 diabetes

Using conventional B-mode, no gross difference was found in the pancreatic echogenicity, homogeneity, contour and capsule sharpness between the studied children with type 1 diabetes and controls. Moreover, no pancreatic focal lesions or ultrasound evidence of pancreatitis was found in any of the subjects.

**Fig. 1:**
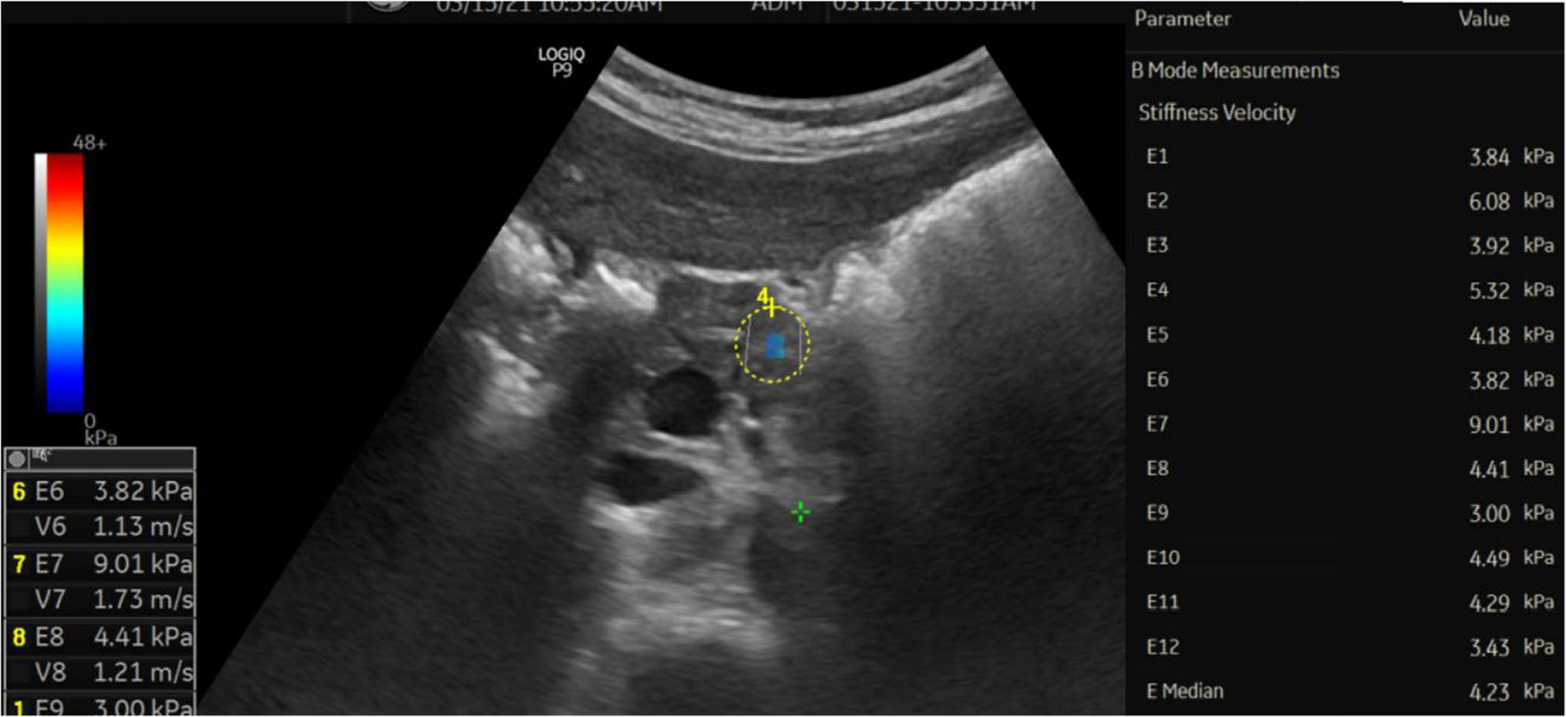
Transverse oblique shear wave elastography in the epigastric region in a 10-year-old girl with recent-onset type 1 diabetes (patient number 7) shows a region of interest in the body of the pancreas with a median pancreatic stiffness of 4.23 Kpa

**Fig. 2:**
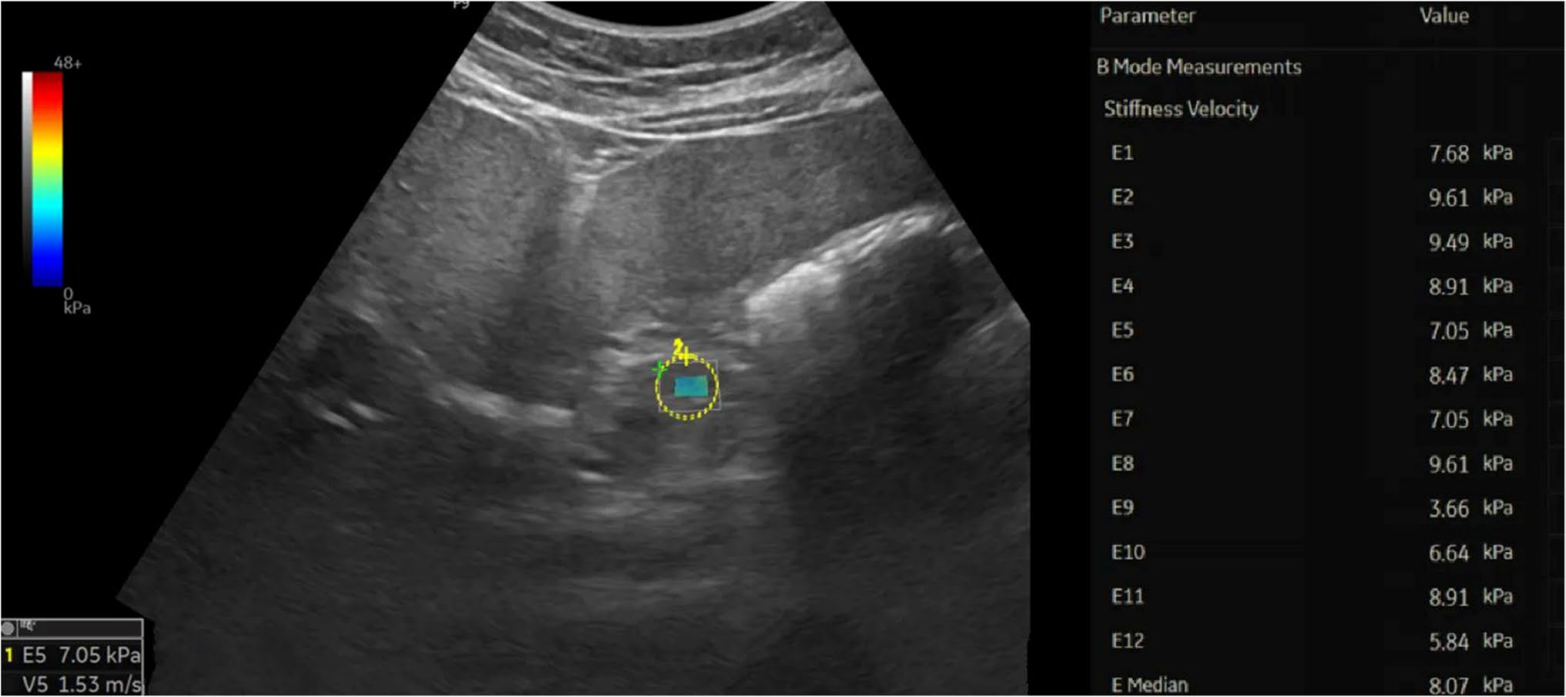
Transverse shear wave elastography in the epigastric region in an 18-year-old woman with long-standing type 1 diabetes (patient number 22) shows a region of interest in the body of the pancreas with a median pancreatic stiffness of 8.07 Kpa

**Fig. 3:**
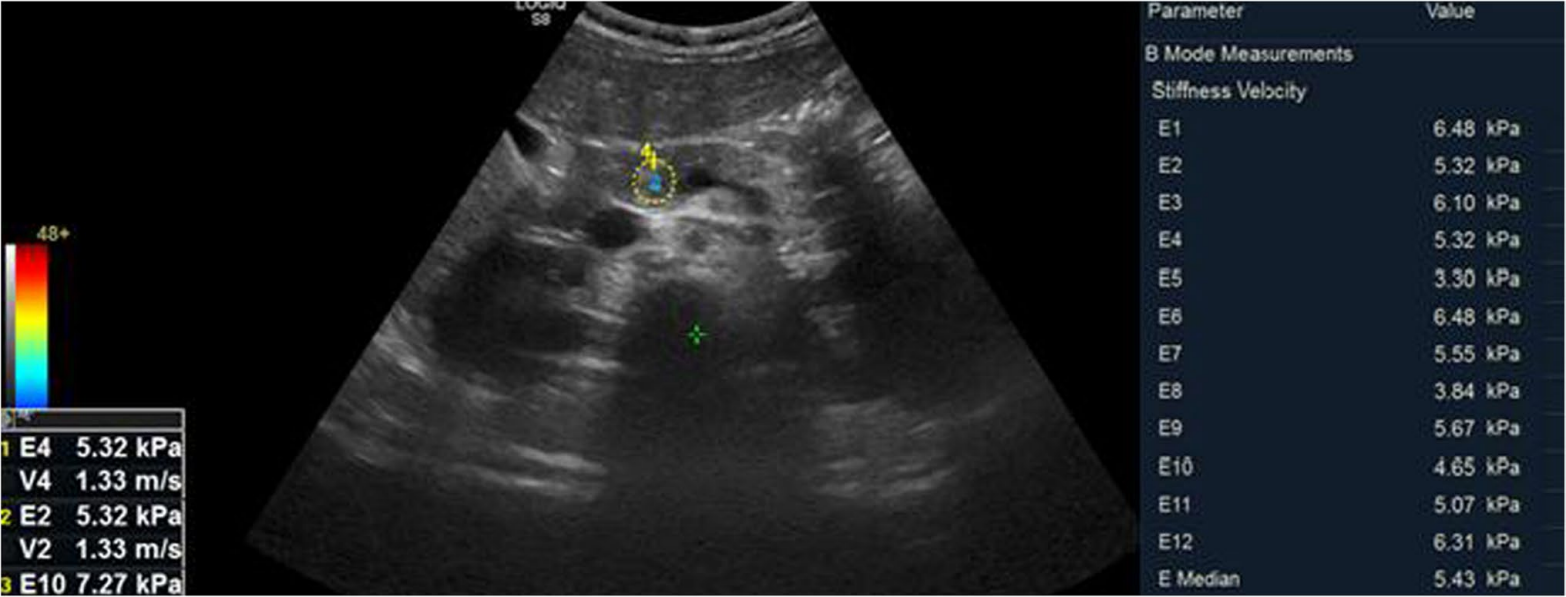
Transverse shear wave elastography in the epigastric region in a healthy 14-year-old boy (control number 4) shows a region of interest in the body of the pancreas with a median pancreatic stiffness of 5.43 Kpa

The mean SWE of the studied children with type 1 diabetes was 5.96±1.65 Kpa (range: 3.87–9.5), while that of the controls was 5.57±0.27 Kpa (range: 5.15–5.92), with no significant difference between the two groups (*P*=0.105). However, upon comparing the SWE between the studied children with recent-onset type 1 diabetes, those with long-standing type 1 diabetes and controls, a significant difference was found between the three groups (*P*<0.001). SWE was found to be highest in those with long-standing type 1 diabetes and lowest in those with recent-onset type 1 diabetes (*P*<0.001) (Table 2, Figs. 1, 2 and 3).

**Table 2:** Comparison between children with recent-onset type 1 diabetes, long-standing type 1 diabetes and controls for various clinic-laboratory and radiologic parameters

		Children with recent-onset type 1 diabetes *n*=25	Children with long-standing type 1 diabetes *n*=25	Controls *n*=50	Test value	*P*-value^*d*^
Age (years)	Mean±SD	12.84±3.54	13.24±3.28	12.08±3.14	1.164^*a*^	0.32
	Range	2–14	10–18	8–11		
Gender	Females	11 (44.0%)	17 (68.0%)	23 (46.0%)	3.882^*b*^	0.14
	Males	14 (56.0%)	8 (32.0%)	27 (54.0%)		
Weight (kg)	Mean±SD	33.00±10.06	50.02±12.97	36.40±4.54	27.70^*a*^	**<0.001**
	Range	13–46	29–70	32–45		
Weight z score	Median (IQR)	−0.05 (−0.26–0.86)	−0.03 (−0.72–0.81)	0.90 (0.55–0.96)	23.534^*c*^	**<0.001**
	Range	−0.9–2.36	−1.26–1.19	0.41–1.33		
Height (cm)	Mean±SD	130.80±16.64	148.12±8.49	141.50±3.53	20.803^*a*^	**<0.001**
	Range	98–158	134–164	137.5–147		
Height z-score	Median (IQR)	−0.24 (−1.22–0.77)	−0.77 (−2.44–0.41)	0.87 (0.73–0.94)	52.098^*c*^	**<0.001**
	Range	−1.82–2.33	−2.64–0.27	0.57–1.82		
BMI	Mean±SD	18.69±2.81	24.47±5.67	18.12±1.41	32.865^*a*^	**<0.001**
	Range	13.5–24.1	18.6–37.1	16.9–20.8		
BMI z-score	Median (IQR)	0.87 (−0.27–1.72)	0.79 (0.36–0.88)	0.65 (0.46–0.71)	1.077^*c*^	0.58
	Range	−2.66–1.89	−0.25–1.93	0.15–1.06		
Shear wave elastography (KPa)	Mean±SD	4.81±0.62	7.10±1.56	5.57±0.27	47.200^*a*^	**<0.001**
	Range	3.87–5.9	4.62–9.5	5.15–5.92		
**Post hoc analysis**						
	**P1**		**P2**		**P3**	
Weight z-score	0.535		**0.001**		**<0.001**	
Height z-score	**0.005**		**<0.001**		**<0.001**	
BMI	**<0.001**		0.485		**<0.001**	
Shear wave elastography (Kpa)	**<0.001**		**<0.001**		**<0.001**	

*BMI* body mass index, *SD* standard deviation

*a*: one-way analysis of variance; *b*: chi-square test; *c*: Kruskall-Wallis test; *d*: *P*-value <0.05 is significant (bold)

P1: Recent-onset versus long-standing type 1 diabetes, P2: Recent-onset type 1 diabetes versus control, P3: Long-standing type 1 diabetes versus control

### Shear wave elastography and diabetes duration

SWE was found to be significantly correlated with age (*P*<0.001) and diabetes duration (*P*<0.001) among the studied children with type 1 diabetes (Table 3). In addition, multivariate logistic regression analysis revealed that SWE was independently related to diabetes duration (*P*<0.001) (Table 4).

**Table 3:** Spearman correlation coefficients for shear wave elastography in children with type 1 diabetes

	Shear wave elastography (KPa)	
	r	*P*-value^*c*^
Age (years)	**0.604**^*a*^	**<0.001**
Disease duration (years)	**0.656**^*a*^	**<0.001**
Weight (kg)	**0.622**^*a*^	**<0.001**
Weight z score	0.240	0.10
Height (cm)	**0.485**^*a*^	**<0.001**
Height z-score	−0.238	0.01
BMI	**0.687**^*a*^	**<0.001**
BMI z-score	**0.305**^*b*^	**0.03**
Insulin dosage (U/kg/day)	0.175	0.23
HbA1C (%)	**0.301**^*b*^	**0.03**
Fasting C-peptide (ng/mL)	**−0.542**^*a*^	**<0.001**
Frequency of severe hypoglycemia (per month)	**0.387** ^*a*^	**0.005**
LDL (mg/dl)	**0.533**^*a*^	**<0.001**
HDL (mg/dl)	0.059	0.68
Cholesterol (mg/dl)	**0.596**^*a*^	**<0.001**
Triglycerides (mg/dl)	−0.194	0.18
UACR (mg/g creatinine)	0.255	0.07

*BMI* body mass index, *HbA1C* glycated hemoglobin, *HDL* high-density lipoproteins, *LDL* low-density lipoproteins, diabetes, *UACR* urinary albumin creatinine ratio

*a*: highly significant; *b*: significant; *c*: *P* value *<*0.05 is significant (bold)

**Table 4:** Multivariate linear regression analysis for factors associated with shear wave elastography among the studied children with type 1 diabetes

	Unstandardized coefficients		Standardized coefficients	t	*P*-value^*a*^
	B	SE	Beta		
(Constant)	−3.032	3.213		−0.944	0.35
Age (years)	0.202	0.172	0.528	1.174	0.25
Disease duration (years)	0.171	0.042	0.484	4.099	**<0.001**
Weight (kg)	−0.006	0.026	−0.051	−0.230	0.82
Height (cm)	0.026	0.028	0.246	0.913	0.37
BMI	0.007	0.033	0.024	0.219	0.83
HbA1C (%)	0.416	0.095	0.499	4.401	**<0.001**
Fasting C-peptide (ng/mL)	3.815	1.254	0.407	3.043	**0.004**
Frequency of severe hypoglycemia/month	0.361	0.116	0.411	3.114	**0.003**
LDL (mg/dl)	−0.019	0.013	−0.296	−1.435	0.16
Cholesterol (mg/dl)	−0.006	0.007	−0.141	−0.879	0.39

*B* unstandardized beta, *BMI* body mass index, *HbA1C* glycated hemoglobin, *LD*L low-density lipoproteins, *SE* standard error for B

*P*-value <0.05 is significant (bold)

### Shear wave elastography and pancreatic reserve

Regarding the relation between SWE and pancreatic reserve, SWE was negatively correlated with fasting C-peptide (*P*<0.001). SWE was independently associated with fasting C-peptide (*P*=0.004) among the studied children with type 1 diabetes.

### Shear wave elastography and diabetes complications

**Fig. 4:**
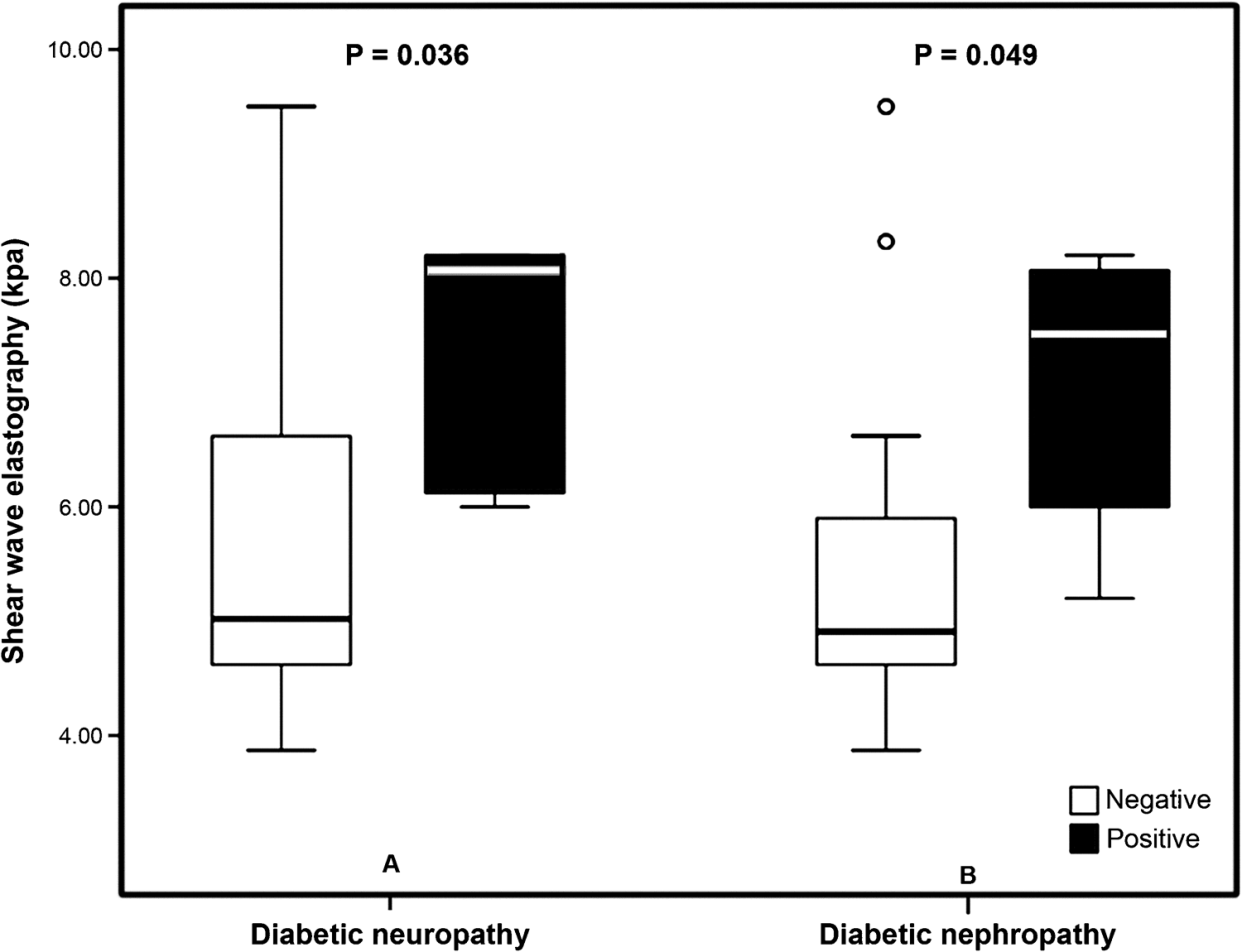
Box plots compare shear wave elastography in children with type 1 diabetes with and without diabetic neuropathy (**a**) and with and without diabetic nephropathy (**b**)

As shown in Table 3, SWE was significantly correlated with the frequency of severe hypoglycemia (*P*=0.005), HbA1C (*P*=0.03), low-density lipoproteins (*P*<0.001) and cholesterol (*P*<0.001). In addition, it was correlated significantly with diabetic neuropathy (*P*=0.04) and with diabetic nephropathy (*P*=0.05) (Fig. 4). Multivariate logistic regression analysis revealed that SWE was independently related to HbA1C (*P*<0.001) and the frequency of severe hypoglycemia (P=0.003) (Table 4).

## Discussion

Studies have described increased SWV due to tissue fibrosis in pancreatic ductal adenocarcinoma [20] and chronic pancreatitis [33]. Recent studies have demonstrated increased SWE of the pancreas in people with type 2 diabetes compared with healthy controls [19]. Regarding type 1 diabetes, studies are scarce and contradictory. One study in children failed to show a significant difference between children with type 1 diabetes and controls [34], in contrast to another study, which did show a difference [30]. In the current study, the mean tissue stiffness of children with type 1 diabetes was not significantly different from that of controls. However, when comparing the tissue stiffness of children with recent-onset type 1 diabetes, long-standing type 1 diabetes and controls, a significant difference was found between the three groups. Children with recent-onset type 1 diabetes had significantly lower pancreatic stiffness while those with long-standing type 1 diabetes had significantly higher stiffness than the controls. This could explain the discrepancy of the results from the previous studies where the duration of diabetes was not considered.

Studies have shown inflammation in the pancreas in the preclinical and early stages of type 1 diabetes [35]. Pancreatic inflammation was observed in recent-onset fulminant type 1 diabetes by dynamic computed tomography (CT) and magnetic resonance imaging (MRI) showing a swollen pancreatic tail and a peripancreatic fluid collection on CT scan and pancreatic swelling with high-intensity areas on MRI [36]. With the progression of diabetes, pancreatic fibrosis usually occurs because of pancreatic inflammation or damage [37]. Chronic hyperglycemia stimulates pancreatic stellate cell activation and proliferation with a subsequent increase of collagen production from these cells, while hypoinsulinemia inhibits acinar cell growth and pancreatic enzyme secretion [38].

Pathological mechanisms associated with tissue edema and inflammation are known to decrease tissue stiffness and SWV [39], while those associated with fibrosis-like chronic pancreatitis increase tissue stiffness and SWV [14, 40]. This could explain the decreased pancreatic stiffness in the studied children with recent-onset type 1 diabetes and increased pancreatic stiffness in the children with long-standing type 1 diabetes and might give insight into early type 1 diabetes diagnosis even in the preclinical insulitis phase.

Long-standing type 1 diabetes leads to pancreatic fibrosis not only in the islets of Langerhans but also in the exocrine pancreatic tissues [41]. In the current study, SWE correlated significantly and independently with diabetes duration. This is in concordance with previous studies on children with type 1 diabetes and people with type 2 diabetes that showed significant correlation between SWE and diabetes duration [30, 42]. In contrast, Püttmann and colleagues [1] didn’t find significant correlation between SWE and the duration of diabetes in people with type 1 diabetes, but their sample size was small (10 men and 5 women).

Type 1 diabetes is characterized by progressive pancreatic islet β-cell loss and dysfunction. During the first stage, only autoantibodies are positive with normal β-cell secretory capacity. Impaired β-cell secretory capacity is first evident in the second stage of diabetes. Symptomatic (stage 3) type 1 diabetes occurs when the functional pancreatic β-cell mass decreases to < 25% of normal. Pancreatic islet β-cell reserve is best estimated by the β-cell secretory capacity of C-peptide [43]. In the current study, SWE was negatively correlated with fasting C-peptide among the studied children with type 1 diabetes. Moreover, multivariate logistic regression for factors associated with SWE revealed that it was independently associated with fasting C-peptide. In line with these results, a previous study on 343 people with type 2 diabetes showed significantly higher SWV in those with low fasting C-peptide suggesting a relation between the pancreatic elasticity and fasting C-peptide among people with type 2 diabetes [44]. Hence, SWE might be a promising radiologic marker together with the fasting C-peptide to monitor disease progression and management from the preclinical stage throughout all the disease stages. Future prospective studies are needed to assess the timing of the SWE changes in relation to the fall in fasting C-peptide.

Interestingly, the frequency of severe hypoglycemia was significantly correlated to SWE among the studied children with type 1 diabetes. Recent studies have demonstrated significant negative correlation between the frequency of hypoglycemia and the fasting C-peptide level among individuals with type 1 diabetes and type 2 diabetes [45, 46]. This agrees with the current study since both SWE and fasting C-peptide are indicators of pancreatic reserve. Thus, SWE is an inexpensive and easy modality that may identify patients with type 1 diabetes who are at increased risk of hypoglycemia.

Glycemic derangement indices (hyperglycemia and hypoglycemia) are associated with increased oxidative stress and inflammatory markers, such as total antioxidant capacity and high-sensitivity C-reactive protein in people with type 1 diabetes [47]. This induces monocyte infiltration, chemokines production and macrophage infiltration eventually leading to tissue damage and fibrosis [48]. In the current study, SWE significantly correlated with HbA1C, low-density lipoproteins and cholesterol among the studied children with type 1 diabetes. In addition, it was significantly related to diabetic neuropathy and nephropathy. In agreement with these results, He et al. [42] found significantly higher SWV among adults with type 2 diabetes having microvascular complications than those without. Moreover, SWV was positively correlated with cholesterol among those having microvascular complications [41]. Similarly, a greater likelihood of developing microvascular complications was independently associated with increasing pancreatic SWV by multivariate linear regression analysis [21]. A possible explanation for increased SWE in those with microvascular complications is that microangiopathy causes pancreatic ischemia, which in turn activates pancreatic stellate cells and induces their proliferation, causing pancreatic fibrosis [49]. Pancreatic fibrosis is known to increase pancreatic SWE [38]. Thus, SWE might be a promising radiologic marker to monitor disease progression and management, identifying those at risk of glycemic derangement and development of microvascular complications.

Even though islet autoantibodies are proven to be related to the possibility of type 1 diabetes development, they are not the primary mediators and do not correlate directly with the degree of β-cell affection. Moreover, the functional B cell reserve in stage 2 and 3 diabetes can’t be diagnosed definitely in vivo because fasting C-peptide is not decreased except late in the disease process. Pancreatic islet cell imaging is not feasible in vivo and pancreatic biopsies are not undertaken due to their invasiveness [50]. Hence, incorporating new modalities like pancreatic SWE with other available investigations may allow for early prediction and mechanistic studies of type 1 diabetes. Moreover, assessing pancreatic SWE changes among people with type 1 diabetes might be clinically helpful for complication risk assessment, early detection, follow-up and management strategy guidance in patients with acute and chronic diabetes. One limitation of this study is its cross-sectional design, which cannot confirm causality. Another limitation is the use of only a single ROI. Hence, further larger longitudinal prospective studies are needed to verify the changes of the SWV during preclinical and different clinical stages of diabetes, further exploring its role in the early detection of diabetes and diabetic microvascular complications.

## Conclusion

Pancreatic SWE changes significantly depending on duration of type 1 diabetes, being lowest in children with recent-onset type 1 diabetes and highest in those with long-standing type 1 diabetes, particularly those with diabetic nephropathy and neuropathy. Hence, SWE might serve as a new assessment tool reflecting diabetic microangiopathy, and therefore could be a promising noninvasive imaging modality for diagnosing and monitoring all phases of type 1 diabetes in children.
